# Phase Field Simulation of Laminated Glass Beam

**DOI:** 10.3390/ma13143218

**Published:** 2020-07-20

**Authors:** Francesco Freddi, Lorenzo Mingazzi

**Affiliations:** Department of Engineering and Architecture, Università di Parma, Parco Area delle Scienze 181/A, I 43124 Parma, Italy; lorenzo.mingazzi@unipr.it

**Keywords:** laminated glass, fracture mechanics, phase-field, delamination, composite

## Abstract

The complex failure mechanisms of glass laminates under in-plane loading conditions is modelled within the framework of phase-field strategy. Laminated glass is widely used for structural purposes due to its safe post-glass-breakage response. In fact, the combination of several glass plies bonded together with polymeric interlayers allows overcoming the brittleness of the glass and to reach a pseudo-ductile response. Moreover, the post-breakage behaviour of the laminate is strictly correlated by the mechanical properties of the constituents. Ruptures may appear as cracks within the layers or delamination of the bonding interface. The global response of a glass laminate, validated against experimental results taken from the literature, is carried out by investigating a simplified layup of two glass plies connected by cohesive interfaces through an interlayer. Delamination of the adhesive interface is described, and crack patterns within the materials are fully described. Finally, the proposed approach put the basis for future comparisons with results of experimental campaign and real-life applications.

## 1. Introduction

Glass has been employed in many forms of art for centuries. Due to its brittle behaviour, its safety is compromised in structural applications if a single glass element is used, as a remedy, laminated glass elements, obtained by coupling together multiple glass plies (float, heat treated or tempered) with polymers or resins interlayer, are widely employed. The failure behaviour of the composite element becomes pseudo-ductile ensuring the safety requirement. In fact, even if one layer is broken, the global stability of the laminate is not compromised as the intact glass plies are still capable of carrying the applied load, and thanks to the interlayer, part of it is transferred to the undamaged areas of the partially broken glass ply.

A vast amount of literature is devoted to the evaluation of the mechanical response of sound laminated glass, both theoretically [1,2,3] and experimentally [4,5,6]. Very few works address the post-breakage response. Extensive tests have been conducted on laminated glass, both for in-plane loading [7,8,9,10] and for out-of-plane loading [11,12], showing its pseudo-ductile failure mechanism. Delamination of the interface has been studied in [13], highlighting the differences between the short and long term response. Fracture investigations of dynamic load have been performed in [14,15].

In order to numerically investigate the failure behaviour of the laminated glass beams, commercial software or numerical models, such as 3D solid, layered shells or smeared models, are usually employed. Different techniques have been used to model the fracture process within the glass plies, such as element erosion/deletion, cohesive zone or extended finite element method, while for the adhesion between glass and interlayer shared node algorithm, penalty-based approaches or intrinsic cohesive modelling are used. However, these techniques might present drawbacks such as complex implementation, mesh dependency or applications to particular specimen with specific parameters. Additionally, only one failure mechanism between rupture of the glass plies and delamination of the interface can be investigated within a single simulation. For more details, see [16].

The phase-field approach seems to be a perfect candidate to capture the failure mechanisms of the laminated glass beam. In fact, the debonding and fracture behaviour of thin brittle films have been described in [17], and the complex failure mechanism of composite materials has been reproduced in [18,19], giving results in good agreement with experimental evidence [20]. Initially proposed in [21], and based on Griffith’s variational approach to fracture, the phase-field method is capable of modelling the nucleation, growth and bifurcation of cracks via the minimisation of an energy functional. Moreover, the Γ-convergence results presented in [22,23,24] provide mathematical robustness. Several formulations of the method have been proposed throughout the years, extending its effectiveness in modelling a wide variety of cases, for example, interpenetration problems [25,26,27], complex mechanical behaviours such as shear fracture [28], ductility [29,30,31,32,33,34], cohesive materials [35] and dynamic analysis [36,37]. Additional details can be found in [38].

Here, a first and novel attempt to numerically model the failure mechanism in laminated glass via phase-field approach is presented. Starting from the formulation proposed in [19] for composite materials, the present model permits to consider, within the same modelling strategy, the damage evolution on each layer and the delamination process of the interface. The mechanical response of the laminate glass is obtained by the minimisation of a two-field energetic functional (displacement and damage fields) defined on the the glass plies. The main advantage of the proposed approach is that the pre- and post-failure behaviours are captured within a single formulation. Normally, the laminated glass models are only developed for pre- or post-breakage behaviour [39]. A simplified layup of a laminated glass with in-plane conditions is considered, whereas a cohesive bilinear law is assumed to model the adhesion between the interlayer and the glass plies. For simplicity, the interlayer viscous behaviour is neglected, as short-term evolution is considered. This approach permits fully characterising the complex failure mechanism of the laminated glass beam giving results which do not suffer from mesh dependence or pre-imposed ad hoc conditions. The influence on the failure mechanism of the mechanical properties of materials is investigated via unidirectional traction tests, whereas a four-point bending test has been studied to compare the numerical results with experimental data.

## 2. Laminated Glass Beam Mechanical Response

### 2.1. Physical Problem

**Figure 1:**
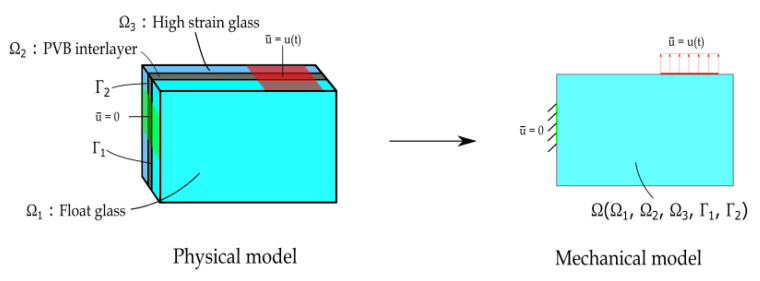
Physical and mechanical problem.

The mechanical response of a representative laminated glass element is studied. The specimen reported in Figure 1 is a hybrid glass–resin laminate composed of three different layers. The first element $\Omega_{1}$ consists of a float glass ply, which presents a low strain elastic brittle behaviour. A second glass ply $\Omega_{3}$, with a higher strain limit compared to $\Omega_{1}$, serves as support after rupture, permitting an overall pseudo-ductile response of the composite glass beam [12]. The central layer $\Omega_{2}$, or interlayer, is made of polyvinyl butyral (PVB) and acts as bonding material between the two glass plies. The layer connection is modelled with two cohesive interfaces: $\Gamma_{1}$ and $\Gamma_{2}$. Certainly, several combinations of different types of glass plies bonded together are commonly adopted in structural applications; however, the results obtained for the considered simple but representative package may be extended to more complex cases.

As the overall composite thickness $h=h_{1}+h_{2}+h_{3}$ is very small compared to the characteristic specimen size, and since fractures in the plies and in the interfaces are parallel to the mid-plane of the specimen, investigation is limited to boundary conditions acting on the mid-plane of each layer as in Figure 1. It is therefore possible to simplify the analysis to an in-plane mechanical regime according to the consideration made in [19]. The investigation of out-of-plane conditions will be considered in a forthcoming paper.

**Figure 2:**
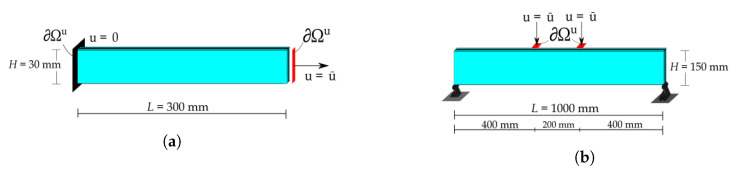
Test set-ups: (**a**) Unidirectional traction test (UD test) set-up. (**b**) Four-point bending test (FPB test) set-up.

Two specific test set-ups will be investigated:

- *Unidirectional traction test* (UD test): We consider the case in which the element is uniaxially loaded orthogonally to the direction of the dominant cracks that may appear within the element. Cracks parallel to the direction of loading are characterised by null or negative opening stress so that they do not alter the tensile response. Therefore, one can only consider the effects of the cracks orthogonal to the tensile stress that can be efficiently modelled in a unidirectional traction test. This simple but representative set-up is optimal to analyse the influence of the material parameters on the mechanical response of the laminated glass element. The traction test has been modelled as the tensile set-up presented in [8] where compression forces on the specimen ends, which might lead to local failure, are avoided by gluing both ends of the specimen to steel angles where bolted plates apply the traction force. The set-up is reported in Figure 2a. The representative element is fixed on the left extremity, whereas a horizontal displacement is applied on the right side. To provide a sufficient redundancy to the laminated glass beam [9], a tempered glass is considered as high strain glass, thus permitting to investigate a wider variety of interface behaviours thanks to its higher loading capacity.
- *Four-point bending test* (FPB test): The four-point bending test reported in [40] is studied. The set-up, shown in Figure 2b, consists of two float glass plies with a SGP interlayer. The purpose of the test is to compare the results obtained from the numerical method with experimental data.

The float glass ply is treated as a brittle material with a well-defined ultimate stress. Accordingly, a phase field formulation based on the Griffith theory of fracture is assumed. For sake of simplicity, the interlayer is treated as linear elastic even if it exhibits a viscous response. Our goal is not to investigate long-term behaviour, but to concentrate on brutal rupture.

When tempered glass is considered as high strain glass, the analysis is terminated as damage initiates in the high strain glass ply.

A cohesive interface with nonlinear behaviour is adopted between two layers. In particular, a bilinear law is considered with an initial elastic loading stage followed by a dissipative phase. Once the limit of the dissipative phase is reached, complete delamination of the interested zones happens leading to debonded areas where there are no interactions between layers.

### 2.2. Problem Formulation: Phase Field Approach

The physical problem has been modelled using the phase field approach for brittle fracture. The basic concepts of the method, such as the energy contributions and the equations governing the problem evolution, which are useful for the comprehension of the paper, are presented. Additional details can be found in [19].

Let Ω be a solid occupying a portion of the space $R^{d}$ (d=1,2,3) with boundary ∂Ω and let the solid be subdivided into $\Omega_{i}$ (i=1,2,3) layers with interfaces $\Gamma_{j}$ (j=1,2) between two subsequent layers. The displacement field of the *i*-th layer is referred as $u_{i}$, and $\delta_{j}=u_{j}-u_{j+1}$ is the relative displacement between two consecutive layers which characterises the mechanical response of the *j*-th interface. The slip norm is also defined as $\delta_{j}=\left|\left|u_{j}-u_{j+1}\right|\right|$. For each *i*-th layer, imposed displacements ${\bar{u}}_{i}$ are applied on the portion of the boundary $\partial \Omega_{i}^{u}$.

Due to the action of the imposed displacement, cracks may appear within the solid. In order to treat the discrete fracture problem with a regularised approach, a scalar field $\alpha \left(x\right):\Omega \to \left[0,1\right]$, which can be seen as a damage parameter, is introduced. The value α=0 is assumed where there the material is intact, while the presence of a crack will be described as a band of non-zero thickness where the scalar field assumes the value α=1, implying a zone with a loss of stiffness and where cohesion is fully lost.

For each layer $\Omega_{i}$, a strain energy density and a fracture energy density are defined:

- strain energy density
  (1) $$\psi_{{}_{i}}\left(u_{i},\alpha_{i}\right)={\left(1-\alpha_{i}\right)}^{2}\frac{1}{2}C_{i}\left[\nabla^{s}u_{i}\right]\cdot \nabla^{s}u_{i}$$
  which represents the quadratic strain energy density of a damageable material with $C_{i}$ being the fourth-order elastic tensor of the material and $\nabla^{s}u_{i}$ being the symmetric part of the displacement gradient.
- fracture energy density
  (2) $$\Psi_{{}_{i}}\left(\alpha_{i}\right)=\frac{\gamma_{i}}{2}\left(\frac{\alpha_{i}}{\ell_{i}}+\ell_{i}{\left|\left|\nabla \alpha_{i}\right|\right|}^{2}\right)$$
  where the constant $\gamma_{i}$ represents the fracture energy of each layer, whereas $\ell_{i}\in R^{+}$ is a length parameter that defines the width of the transition zone between the damaged and the undamaged zone in the i-th layer. A parameter representative of the material strength is defined as $w_{i}=3\sqrt{2}\gamma_{i}/8\ell_{i}$.

**Figure 3:**
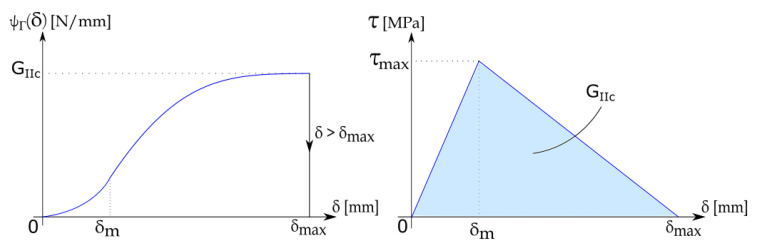
Interface internal potential energy and bilinear τ−δ diagram used in simulations.

For the *j*-th interface, the following internal potential energy (see Figure 3) is defined,

(3) $$\psi_{\Gamma_{j}}\left(\delta_{j}\right)=\left\{\begin{array}{l} \int_{0}^{\delta_{m}}K_{l_{j}}\delta_{j}\;d\delta \;,\;\mathrm{if}\;\delta_{j}\leq \delta_{m}\\ \frac{1}{2}K_{l_{j}}\delta_{m}+\int_{\delta_{m}}^{\delta_{max}}K_{d_{j}}\delta_{j}\;d\delta \;,\;\mathrm{if}\;\delta_{m}<\delta_{j}\leq \delta_{max}\\ 0\;,\;\mathrm{if}\;\delta_{j}>\delta_{max} \end{array}\right.$$

where $K_{l_{j}}$ is the slope of the loading phase of the interface, $K_{d_{j}}$ is the slope of the dissipative phase of the interface, $\delta_{m}$ is the slip between two different layers associated with the maximum stress $\tau_{max}$ and $\delta_{max}$ is the slip limit after which delamination starts. The stress–slip diagram has been obtained as

(4) $$\tau_{j}\left(\delta_{j}\right)=\partial_{\delta_{j}}\psi_{\Gamma_{j}}\left(\delta_{j}\right)=\left\{\begin{array}{c} K_{l_{j}}\delta_{j}\;,\;\mathrm{if}\;\left|\delta_{j}\right|\leq \delta_{m}\\ \mathrm{sign}\left(\delta_{j}\right)\left(\frac{|\delta_{j}|-\delta_{max}}{\delta_{m}-\delta_{max}}\right)\tau_{max}\;,\;\mathrm{if}\;\delta_{m}<\left|\delta_{j}\right|\leq \delta_{max}\\ 0\;,\;\mathrm{if}\;|\delta_{j}|>\delta_{max} \end{array}\right.$$

The expression of the total energy functional becomes

(5) $$\Pi_{l}\left(u,\delta ,\alpha \right)=\sum_{i=1}^{3}\int_{\Omega_{i}}\left(\psi_{{}_{i}}\left(u_{i},\alpha_{i}\right)+\Psi_{{}_{i}}\left(\alpha_{i}\right)\right)\;dx+\sum_{j=1}^{2}\int_{\Gamma_{j}}\psi_{\Gamma_{j}}\left(\delta_{j}\right)\;dx$$

The solution of the mechanical problem can be obtained as the minimisation of functional (5) coupled with proper boundary conditions for the displacement as well as additional boundary conditions for the damage field, added to prevent the formation of damage zones near the boundary.

Moreover, an irreversibility condition for the time evolution of the damage field is added to prevent material healing:

(6) $${\dot{\alpha }}_{i}\geq 0\;.$$

The evolution of the problem is described by a set of Euler–Lagrange equations, which are obtained differentiating the functional with respect to the displacement field and the phase field.

(7) $$\left\{\begin{array}{c} \mathrm{div}\;T_{i}-1^{\left(i+j-1\right)}\;\tau_{j}\left(\delta_{j}\right)=0\;\mathrm{in}\;\Omega_{i},\\ \frac{\gamma_{i}}{2\ell_{i}}-\gamma_{i}\ell_{i}\Delta \alpha_{i}+\frac{\partial \psi_{{}_{i}}}{\partial \alpha_{i}}=0\;\mathrm{in}\;\Omega_{i}\;, \end{array}\right.$$

with $T_{i}=\frac{\partial \psi_{{}_{i}}}{\partial \nabla^{s}u_{i}}$ being the Cauchy stress tensor and $\frac{\partial \psi_{{}_{i}}}{\partial \alpha_{i}}$ being the crack driving force of the *i*-th layer. The stresses transmitted at the interface level are $\tau_{j}\left(\delta_{j}\right)=\frac{\partial \psi_{\Gamma_{i}}}{\partial \delta_{j}}n_{j}$ with $n_{j}=\delta_{j}/\left|\left|\delta_{j}\right|\right|$. If the irreversibility condition is taken into account and added into the phase field Euler–Lagrange equation, the following Karush–Kuhn–Tucker conditions, which drive the evolution of the phase field problem, are obtained.

(8) $$\left\{\begin{array}{c} \frac{\gamma_{i}}{2\ell_{i}}-\gamma_{i}\ell_{i}\Delta \alpha_{i}+\frac{\partial \psi_{{}_{i}}}{\partial \alpha_{i}}\leq 0\;\mathrm{in}\;\Omega_{i}\;,\\ \dot{\alpha_{i}}\geq 0\;,\\ \left(\frac{\gamma_{i}}{2\ell_{i}}-\gamma_{i}\ell_{i}\Delta \alpha_{i}+\frac{\partial \psi_{{}_{i}}}{\partial \alpha_{i}}\right)\dot{\alpha_{i}}=0 \end{array}\right.$$

The asymmetric behaviour of glass in traction and compression has been modelled in the four-point bending test by considering an alternative expression for the strain density energy of (1) based on spectral decomposition of the strain tensor. Further details can be found in [26].

## 3. Materials and Methods

The unidirectional traction tests were performed on a representative domain with dimension *L* = 300 mm and *H* = 30 mm, while in the four-point bending test a domain with *L* = 1000 mm and *H* = 150 mm is considered. For all the tests, an unstructured mesh of triangular elements with ratio h/ℓ=1/4, where *h* is the element size, has been used. The maximum tensile strength values of the glass layers are taken from the work in [41]. Rupture in the PVB and SGP layers is excluded so the parameters that govern the failure of the material are not considered. In fact, damage in the interlayer occurs at very high strain level, whereas fracture of glasses initiate at low strain values; therefore, α=0 has been assumed for both the PVB and SGP. In Table 1, the adopted material parameters are reported.

The interface reference parameters are taken from the work in [10] and are listed in Table 2.

First, the unidirectional traction test set-ups are presented in order to provide a better understanding of the failure behaviour and to determine the influence of material and interface parameters.

The microscopic effect of imperfection on a macroscopic scale has been added with the Weibull’s distribution through a scalar field defined on a domain portion with a size of $h_{Weib}=3h$. Accordingly, the surface energy density defined in (2) is modified, leading to weaker and stronger areas. The local resistance constant $w_{i}^{*}$ is obtained as

(9) $$w_{i}^{*}=w_{i}{\left(\ln\left(\frac{1}{1-\lambda }\right)\right)}^{1/m}=\;w_{i}\cdot s.r.f.$$

where λ is a random variable ranging between 0 and 1 and s.r.f. (stress reduction factor) is the coefficients of the scalar field obtained from the Weibull distribution. To model different levels of imperfections in the glass plies, different values of the Weibull’s module *m* have been used. In fact, smaller values return a wider distribution simulating a glass ply with many imperfections, while higher values return a narrow distribution, simulating a more uniform glass ply.

With the introduction of the Weibull distribution, the value of the actual strength in the first layer is given by

(10) $$\sigma_{i}^{*}=\sqrt{E_{i}\cdot w_{i}^{*}}$$

The maximum and minimum values of the actual strength in the first layer are listed in Table 3.

In addition to the Weibull’s modulus, the influence on the failure behaviour determined by different values of the interlayer and interface parameters is also investigated. First, an increment of the Young module of the interlayer is considered, E2=1500 MPa. Eventually, different values of the shear stress transferred between layers are assumed: $\tau_{max}\in \left\{2.5,7.5\right\}$ [MPa]. Finally, different softening slopes of the cohesive interface law are adopted: $\delta_{max}\in \left\{2\delta_{m},6\delta_{m}\right\}$. In Table 4, the unidirectional traction tests list is reported.

Once the differences in the failure mechanism are presented, the focus is put on the four-point bending test, comparing the results from the numerical model with the experimental data provided in [40].

All the tests have been performed through the ad hoc developed python code, which is based on the FEniCS Project open source finite-element library [42]. The result visualisation has been done using the open source data analysis and visualisation application ParaView [43] from Kitware Inc., Los Alamos, NM, USA.

## 4. Results

### 4.1. Unidirectional Traction Tests

#### 4.1.1. Reference Test

First, the UD reference test is presented in order to analyse the mechanical behaviour of the laminated glass beam and to highlight the different phases that characterise the failure process of the composite material.

**Figure 4:**
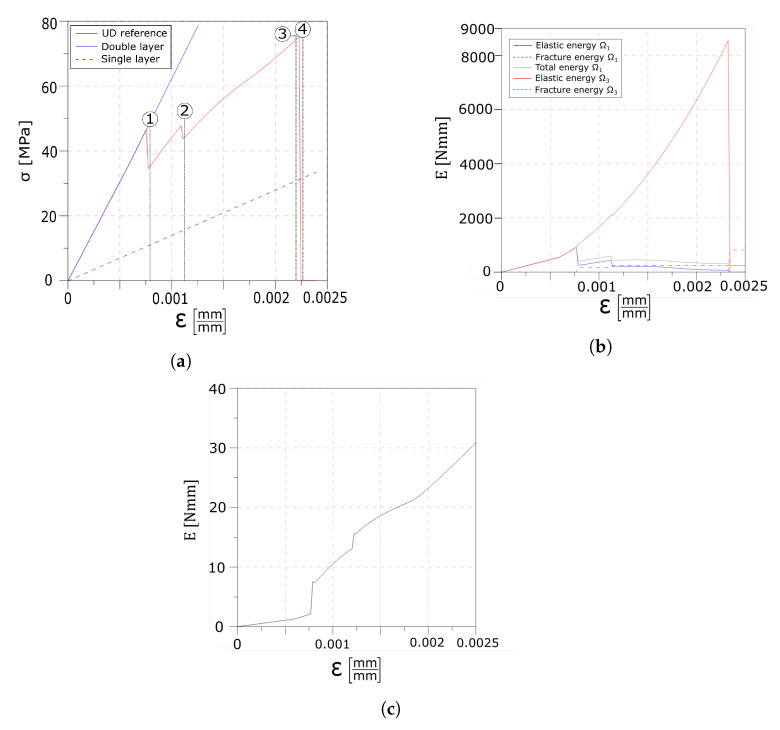
Numerical results of the unidirectional traction (UD) reference test: global response. (**a**) Global stress–strain response of the laminated glass beam. (**b**) Global evolution of the energy contributions. (**c**) Elastic energy contribution of the interlayer.

An initial homogeneous linear elastic stage is shown until the float glass strain limit is reached (point 1 in Figure 4a), and the first crack set in the first layer appears, which is evidenced by a sudden drop in the global stress–strain response. A discontinuity can also be observed in the evolution of the elastic and fracture energy values of the float glass, as reported in Figure 4b. The linear slopes obtainable with one and two layers are added in Figure 4a for comparison. The position and the value of the strain at which the first crack set appears are also dependent on the imperfections modelled via the Weibull’s distribution. This can be better appreciated by comparing the results with the ones presented in Section 4.1.2. At this point, the interface starts to transfer the load from the tempered glass to the undamaged part of the float glass layer via the interlayer. By further increasing the applied strain, the tensile strength of the float glass ply is reached again and a second set of cracks, which is usually placed on the opposite side of the specimen compared to the position of the one already formed, develops in the first layer. The formation of the new crack set is shown by the second drop in the stress–strain response (point 2 in Figure 4a) as well as in the energy plot. An additional increment of the applied displacement starts the delamination process as the relative displacement between the first layer and the interlayer reaches its maximum value. The delaminated areas, which are positioned next to the cracks, keep increasing until ultimate failure of the composite beam is reached and the rupture initiates in the tempered glass ply (point 5 in Figure 4a). Figure 4c reports the elastic energy of the interlayer, however these values are much lower compared to the ones of the glass layers (approximately two orders of magnitude).

**Figure 5:**
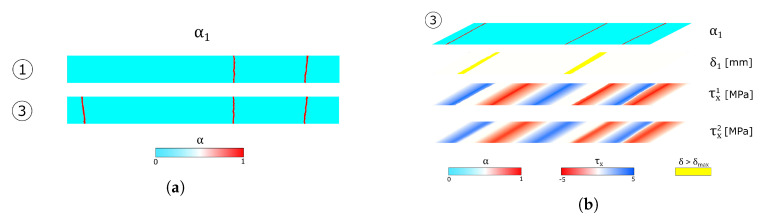
Numerical results of the UD reference test: crack evolution overview. (**a**) Crack pattern in the first layer. (**b**) Exploded view of the composite glass beam.

The crack pattern in the first layer at the instants reported in the circled number is shown in Figure 5a, while the exploded view of the laminated glass is shown in Figure 5b.

**Figure 6:**
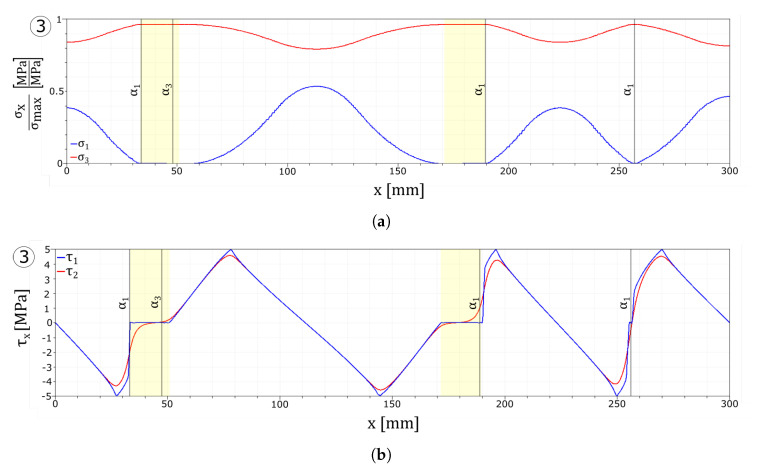
Numerical results of the UD reference test: stresses plot. (**a**) Glass plies stress profiles. (**b**) Interface stress profiles.

Figure 6 shows stress values in the glass plies normalised with respect to the maximum tensile strength of the corresponding layer and the stress profiles along the interfaces. The yellow highlighted zones correspond to the delaminated areas in the first interface, while vertical lines are placed in correspondence with the crack positions for each layer. Here, the mechanical behaviour of the laminated glass beam can be better appreciated. In particular, Figure 6a shows that the presence of cracks in the first layer leads to zones in the tempered glass characterised by high stress values. Moreover, from Figure 6b, it can be noticed that in these glass portions delamination is also present and the interface stresses are null. In fact, the relative displacement is higher than $\delta_{max}$ and no load is transferred to the first layer. Far from the delaminated zones, the interlayer permits the transfer of a portion of the load between the two glass plies, as the interfaces stress profiles follow a similar path along the beam length.

#### 4.1.2. Weibull’s Module Tests

Here, the uniaxial test has been performed with Weibull’s module m=9 in order to simulate a float glass ply with many irregularities, and therefore with high difference between the minimum and maximum tensile resistance values of the layer. Eventually, the mechanical response of a glass with a low level of imperfection has been modelled by increasing the Weibull’s modulus value to m=52.

**Figure 7:**
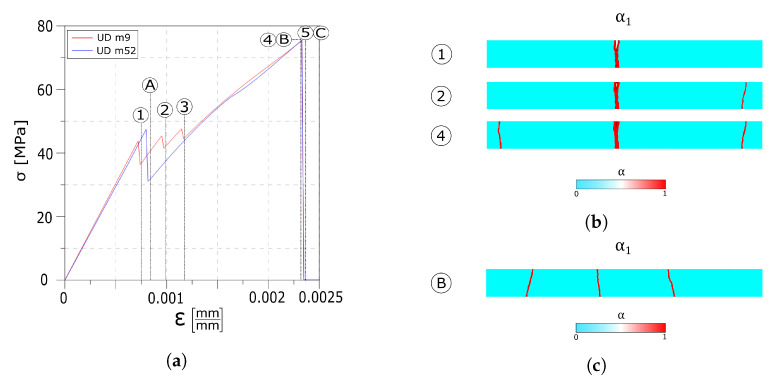
Numerical results: (**a**) Global stress–strain response. (**b**) UD m9 crack evolution overview in the first layer. (**c**) UD m52 crack evolution overview in the first layer.

As shown by in the stress–strain diagram in Figure 7a, a wider Weibull distribution affects the strain value at which the first crack is formed (point 1 and point A) as well as the timing at which each crack set is formed. In the UD m52 test, all the cracks nucleate at the same instant, while in the UD m9 test, the cracks are formed at different moments. Each crack is formed instantaneously as can be seen by the three (particularly one) drops in the stress–strain curve. Once the cracks are formed, by further increasing the strain, delamination between the float glass ply and the interlayer begins, as evidenced by the change in slope of the stress–strain curve in its final segment. As in the previous test, the delaminated areas are close to the fractures in the first layer.

The snapshots of the crack pattern in the first layer for the UD m9 test at the instants reported in the circled number are shown in Figure 7b; here, the three crack sets nucleate at different instants. Figure 7c shows the crack pattern of the UD m52 test.

**Figure 8:**
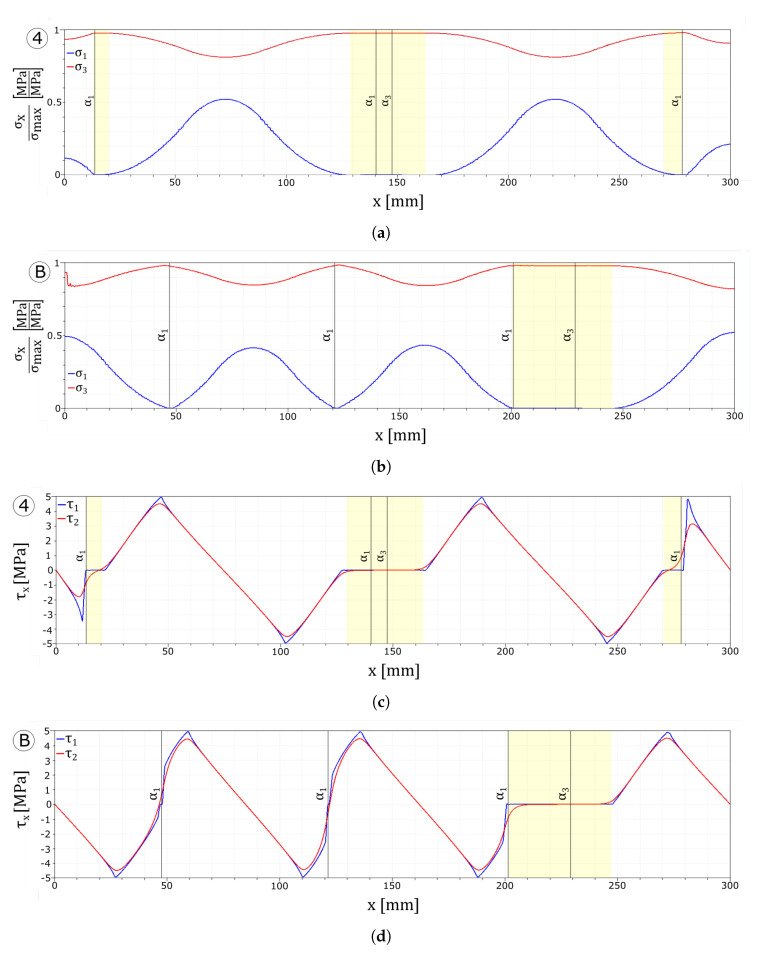
Numerical results: stress plots. (**a**) UD m9 glass plies stress profiles. (**b**) UD m52 glass plies stress profiles. (**c**) UD m9 interface stress profiles. (**d**) UD m52 interface stress profiles.

Figure 8a,b reports the stresses in the glass layers normalised with respect to their tensile strength. The interface stress values, reported in Figure 8c,d, as in the previous simulation, vanish in the delaminated areas close to the cracked zones and transfer load to the undamaged parts of the float glass ply.

#### 4.1.3. Interlayer Stiffness Test

**Figure 9:**
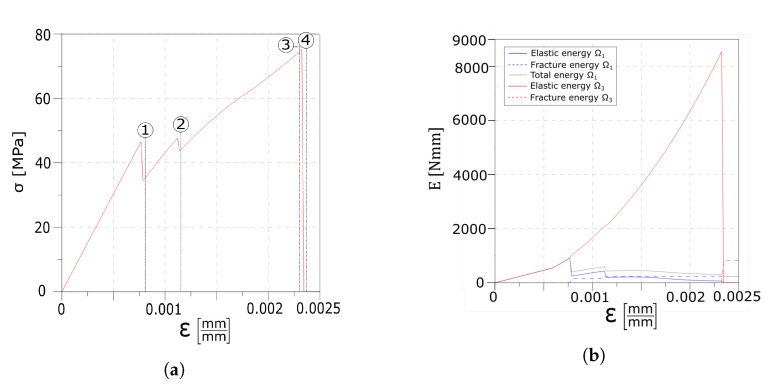
Numerical results of the UD E2 1500 test: global response. (**a**) Global stress–strain response of the laminated glass beam. (**b**) Global evolution of the energy contributions.

A stiffer interlayer with elastic modulus $E_{2}=1500$ MPa is considered. From Figure 9a,b, no noticeable differences are outlined compared to the UD reference test. Two minor differences are the higher values of the interface elastic energy and a slightly larger delamination zone between the float glass ply and the interlayer. For brevity, no figure is reported.

#### 4.1.4. Weak Interface Tests

A weaker interface is now considered, first with maximum stress value equal to $\tau_{max}=2.5$ MPa and eventually with the ultimate displacement limit value of the dissipative phase of the interface set to $\delta_{max}=2\delta_{m}=0.06$ mm.

**Figure 10:**
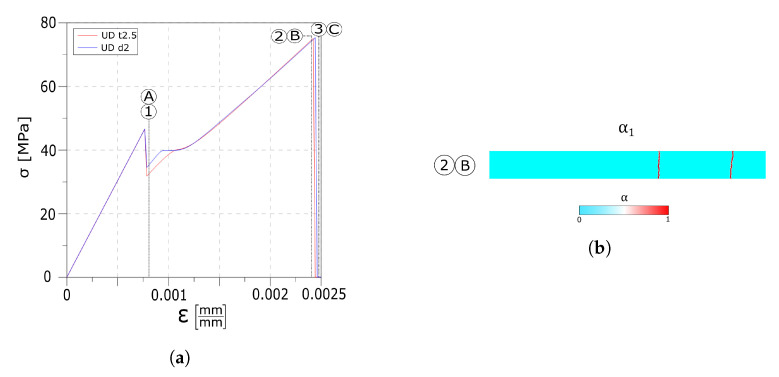
Numerical results. (**a**) Global stress–strain response. (**b**) UD t2.5 and UD d2 crack overview in the first layer.

Here, both tests present a similar behaviour as evidenced from Figure 10 and a single set of cracks is formed in the float glass ply. As the same Weibull’s module has been used for both the tests (m=25), the two crack patterns coincide. As the strain increases, the slope of the curves changes rapidly, denoting the delamination process which continues until damage is initiated in the tempered glass ply.

**Figure 11:**
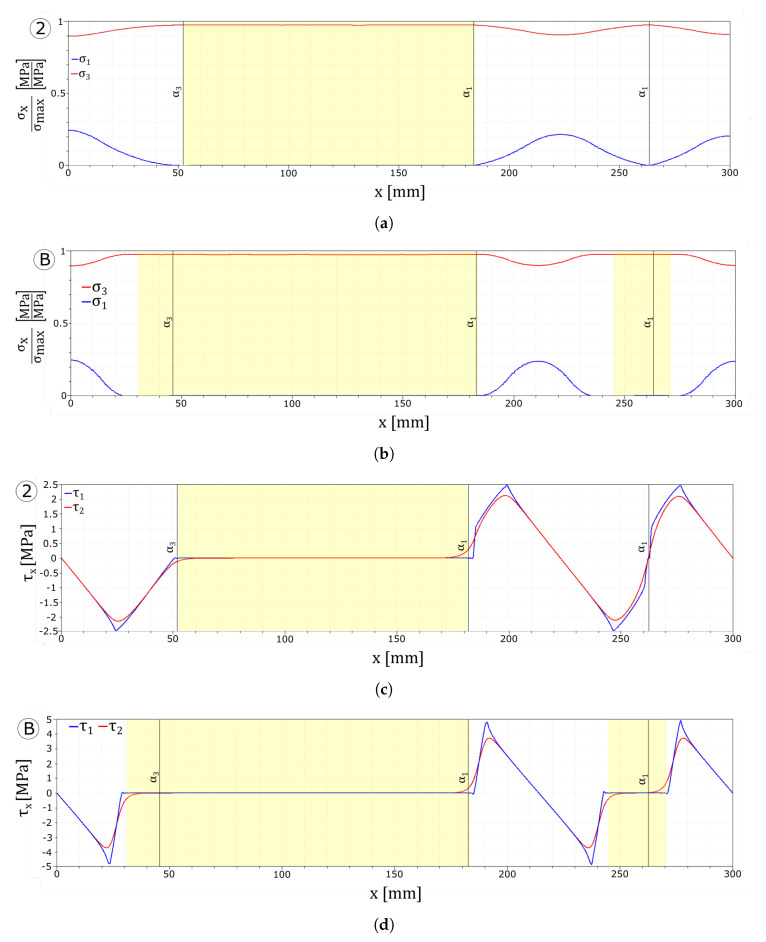
Numerical results: stress plots. (**a**) UD tau2.5 glass plies stress profiles. (**b**) UD d2 glass plies stress profiles. (**c**) UD tau2.5 interface stress profiles. (**d**) UD d2 interface stress profiles.

Figure 11 shows the stresses in the glass plies and in the interfaces. In both tests, due to the weaker interface, dissipation starts earlier and the delaminated area affects more than half of the beam length.

#### 4.1.5. Strong Interface Tests

A stronger interface is now considered. First, the maximum stress has been set equal to $\tau_{max}=7.5$ MPa. After, the limit value of the dissipative phase of the interface has been increased to $\delta_{max}=6\delta_{m}=0.18$ mm.

**Figure 12:**
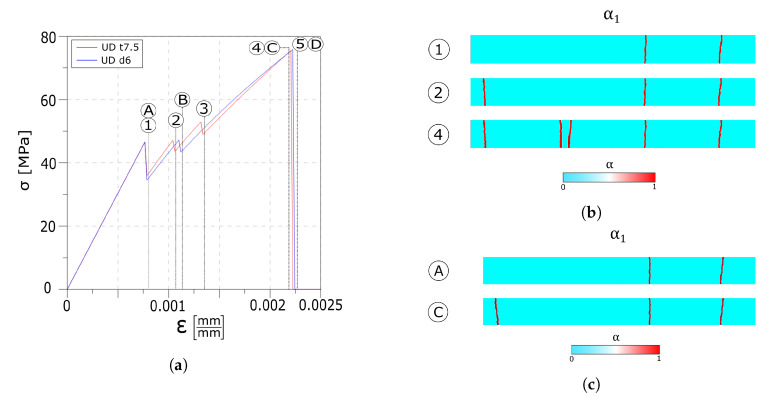
Numerical results: (**a**) Global stress–strain response. (**b**) UD t7.5 crack evolution overview in the first layer. (**c**) UD d6 crack evolution overview in the first layer.

The overall stress–strain responses of Figure 12a show a similar behaviour compared to the one observed in the reference value test up to point 1. Increasing the strain leads to the formation of the second crack set (point 2 and point B). Once the second crack set is formed, in the UD tau7.5 test, the higher value of the interface maximum stress allows the formation of a third crack set, as shown by the third drop in the stress diagram (point 3 in Figure 12a). The linear path of the diagram, up to complete failure in both the curves, indicates that no delamination of the interfaces takes place.

Figure 12b reports the crack patterns snapshots at different instants in the float glass ply.

**Figure 13:**
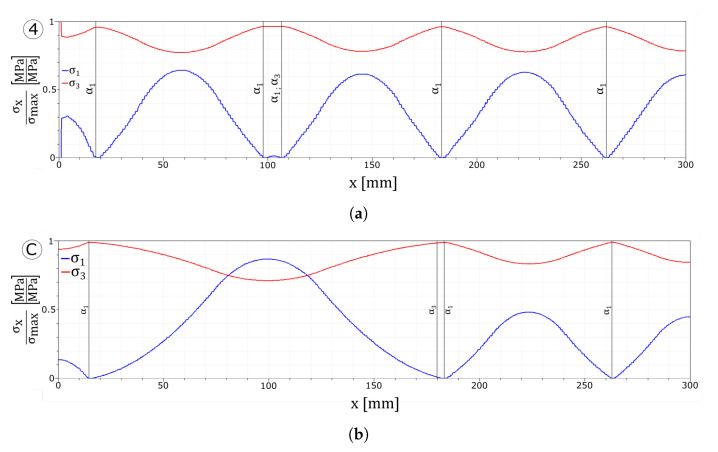

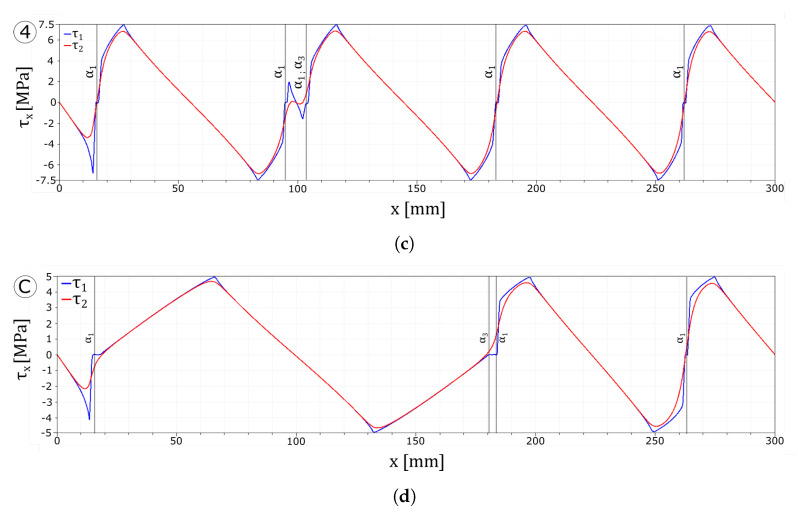
Numerical results: stress plots. (**a**) UD tau7.5 glass plies stress profiles. (**b**) UD d6 glass plies stress profiles. (**c**) UD tau7.5 interface stress profiles. (**d**) UD d6 interface stress profiles.

The tensile stresses in the glass plies normalised with respect to the maximum tensile strength of the corresponding layer, and the interface stresses are reported in Figure 13. All the plots refer to the instant before the damage initiates in the tempered glass ply.

In Figure 13a,c, it can be seen how the higher value of the maximum stress allows the interface to transfer the load from one layer to the other in a more effective way. The absence of delamination permits loading all of the undamaged zones of the float glass ply instead of only the areas which are far from the cracks.

Figure 13b shows that a higher limit value of the dissipative phase of the interface does not permit to transfer enough load from the tempered glass to the float glass ply to create an additional crack in the first layer. Nonetheless, as can be seen from Figure 13d, the larger dissipative phase avoids delamination of the interfaces.

### 4.2. Four-Point Bending Tests

**Figure 14:**
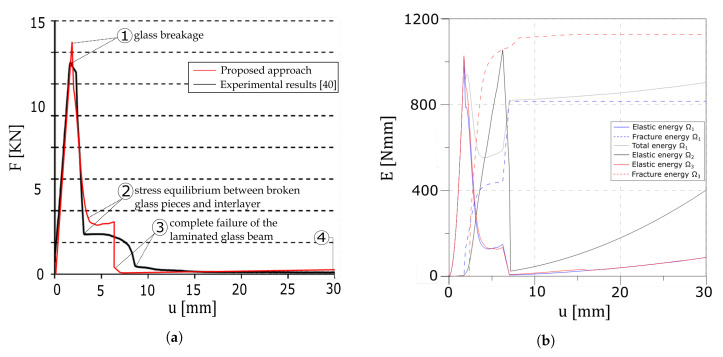
Numerical results of the four point bending test. (**a**) Force–displacement diagram of the proposed approach and experimental result taken from Figure 9 of the work in [40]. (**b**) Global evolution of the energy contributions.

Here, the results of the four-point bending test are presented and compared to the experimental evidence illustrated in [40]. Figure 14a reports the load–displacement curve plot, whereas the energetic contribution of each layer is drawn in Figure 14b.

An initial linear homogeneous response is obtained until the maximum resistance in the glass plies is reached (point 1). Here, one crack appears within the first layer, followed by the formation of another fracture in the second glass ply. As cracks are formed on both the glass layers (point 2), the interlayer creates an equilibrium state between the two broken glass ply through the interfaces. Delamination starts until cracks fully develop in both the glass layers (point 3). The global response is in good agreement with the experimental results of the work in [40].

**Figure 15:**
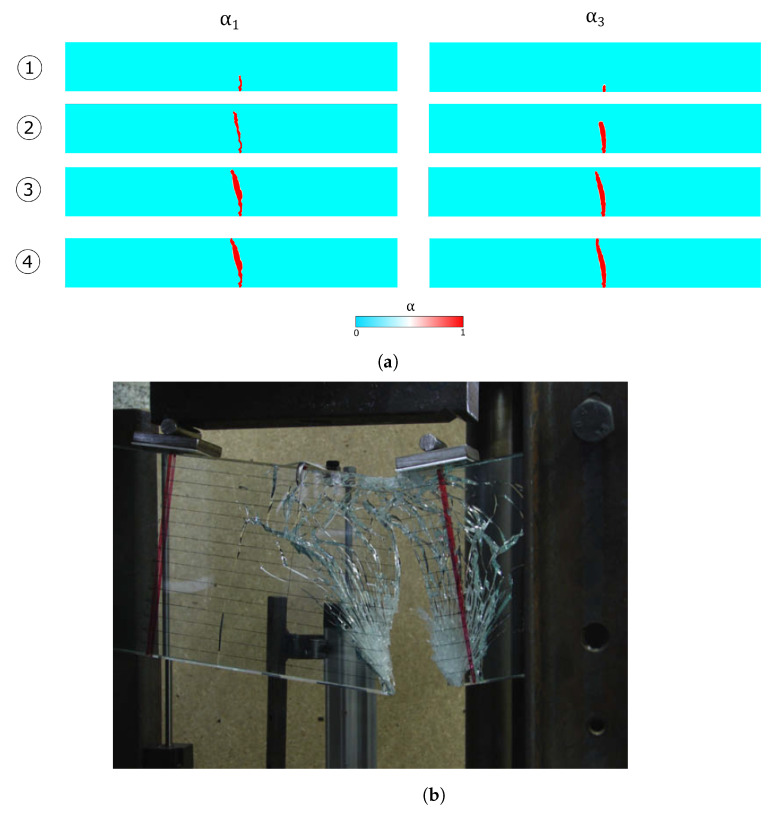
(**a**) Snapshots of the crack pattern at the instants reported in the circles. (**b**) Figure 10 from the work in [40], showing an example of fissured laminated glass beam.

Figure 15a reports the development of the crack pattern as the applied load increases, showing the crack evolution though time. It is possible to observe that the numerical results are able to capture the fissure obtained in the laminated glass beam shown in Figure 15b.

## 5. Discussion

The adopted phase field approach reproduces the failure mechanisms of laminated glass beam. Additionally, the proposed approach enables separate investigation of the energetic contributions of each dissipative phenomenon.

The reference test illustrates the pseudo-ductile response that develops within the laminated glass beam. Multiple crack sets are formed in the float glass ply, while, at the interface level, the appearance of cracks is reflected as jumps in the stress profiles. Moreover, the increase in the relative displacements between layers leads to the progressive delamination of the interface, before the tempered glass reaches its ultimate capacity. Figure 4a allows the comparison of the stress–strain response of the partially broken laminated glass with the curve of intact double and single layer specimens. Comparing the stress diagrams in Figure 6 shows how the interface is able to transfer the load from the third to the first layer.

The variation of the Weibull’s module shows how the presence of imperfections within the glass plies heavily impact the failure behaviour of the composite material. In fact, a higher level of imperfection leads to the formation of several cracks at different times due to modifications in the tensile resistance within the float glass ply. On the contrary, when a higher Weibull’s module value has been used, all the cracks nucleates at the same instant, reflecting the more homogeneous tensile resistance of the glass ply. The failure behaviour of the composite glass beam is therefore characterised by a two-step brittle fracture mechanism: one for the float glass and one for the tempered glass, divided by a single delamination process.

Similarly to the work in [7], changes in the interlayer stiffness do not produce noticeable differences in the response of the laminated glass beams. In fact, results similar to the unidirectional traction reference tests have been obtained, both in terms of stress–strain response as well as in the developed crack pattern.

Two different weak interfaces have been investigated, initially a lower maximum stress is assumed, while in the second case a smaller dissipative phase is considered. Compared to the reference test, the weaker interface causes an earlier delamination phenomenon and no additional cracks are formed. Moreover, comparing the two curves in Figure 10a and the stress profiles in Figure 11, a smaller dissipative phase lead to an earlier and widespread delamination phenomena than in the case with a lower maximum stress.

When stronger interfaces are considered, no delamination develops between the first layer and the interlayer, while in the case of higher value of the maximum interface stress, an additional crack set is formed. Similarly to the weak interfaces tests, the drop value in the stress–strain response is influenced by the interface parameters. In fact, stiffer interfaces lead to a smaller jump in the stress curve, whereas softer interfaces lead to a higher jump (point 1/A and point 2/B in Figure 12a and point 1/A in Figure 10a). Comparing Figure 13 with Figure 6 and Figure 11 evidences how a stronger interface is capable to transfer the load more effectively between the two glass plies, whereas for equispaced cracks, higher stresses can be achieved in the undamaged zones of the broken float glass plies and delamination is avoided.

As seen in the previous tests, the delamination phase develops after the interface reaches its limit capacity and no additional cracks nucleates in the first layer. The absence of this dissipation phenomenon might give rise to safety issues as there are no other visible indications of the pseudo-ductile process approaching its ultimate limit.

The four-point bending test has been considered to compare the numerical results with experimental data. The mechanical response of the laminated glass beam is captured correctly, as seen from Figure 14. The differences in the late softening branch are probably due to the absence of viscous behaviour of the interlayer in the numerical modelling strategy. The analysis of the energetic contributions in Figure 14b offers an interesting insight in the dissipation phenomena. In fact, once a crack develops, the fracture energy of the glass plies increases whereas the elastic energies drop; the interlayer activates and the elastic energy significantly increases until delamination of the interfaces starts. Nonetheless, the fissure profile developed in the first and third layers is captured correctly as evidenced in Figure 15.

## 6. Conclusions

In order to predict and understand the complex behaviour of laminate glass, numerical simulations are perfect candidates. In fact, they permit describing the different dissipative rupture mechanisms that are consequences of a large range of material and interface parameters.

The proposed phase field approach appeared to correctly describe the complex nonlinear dissipative mechanisms which characterise the failure mechanism of the laminated glass beams, giving results in good agreement with the literature. In particular, with such approach, the crack distribution and density within each material can be accurately determined, independently from the mesh structure and without the need to introduce predefined crack loci. Indeed, the energetic investigation has permitted determination of the dissipative contribution of the different failure mechanisms which could be of help to design fail-safe laminate glass elements.

Focus has been put on influence of the material parameters. Changes in the Weibull’s module value generate various degrees of imperfection that affect the failure mechanism of the laminated glass beam. Different values of the interfaces parameters showed how the delamination phenomena, and therefore the possibility to transfer load between layers, heavily depends from the interface fracture energy and from the cohesive law as well. Variations of the maximum interface stress or to the slope of the dissipative phase led to different mechanical responses of the laminated glass beam. The good agreement between the results obtained in the four-point bending test with experimental data underlined the capacities of the method; however, full characterisation of the viscous behaviour of the interlayer is required to capture the complete rupture of the laminated glass beam.

This paper represents a first attempt to numerically reproduce the failure mechanisms of laminate glass. Future developments focus on the extension of the proposed model to consider out-of-plane loads and to model more complex scenarios [44,45,46]. Deeper comparison with experimental evidences will permit to calibrate the approach to catch post-failure behaviour of real-life structural elements.

## Figures and Tables

**Table 1:** Materials parameters.

Layer	h [mm]	E [MPa]	ν	*ℓ* [mm]	$\sigma_{\max}$ [MPa]	*m*
1—Float glass UD	5	70,000	0.2	6	56.12 (w = 0.045)	25
2—PVB interlayer	1.52	500	0.45	/	/	/
3—High strain glass UD	5	70,000	0.2	6	177.48 (w = 0.45)	25
4—Float glass FPB	6	70,000	0.2	20	30.91 (w = 0.013)	25
5—SGP interlayer	1.52	580	0.45	/	/	/

h = thickness of the layer; E = Young’s modulus and; ν = Poisson’s modulus; *ℓ* = internal length; $\sigma_{max}$ = maximum tensile strength; *m* = Weibull’s modulus.

**Table 2:** Interface parameters.

$\tau_{\max}$ [MPa]	$\delta_{m}$ [mm]	$\delta_{\max}$ [mm]	$G_{\mathrm{IIc}}$ [N/mm]
5	0.03	$4\delta_{m}$	0.3

**Table 3:** Weibull parameters.

m	s.r.f. min	s.r.f. max	$\sigma_{\min}$ [MPa]	$\sigma_{\max}$ [MPa]
9	0.20	1.62	31.24	67.58
25	0.62	1.16	44.19	60.45
52	0.80	1.075	50.20	58.19

**Table 4:** Unidirectional tests summary.

Test n	Changed Parameter	Value
1—UD reference	/	Reference value
2—UD m9	*m*	9
3—UD m52	*m*	52
4—UD E1500	$E_{2}$	1500 MPa
5—UD tau2.5	$\tau_{max}$	2.5 MPa
6—UD tau7.5	$\tau_{max}$	7.5 MPa
7—UD d2	$\delta_{max}$	$2\delta_{m}$
8—UD d6	$\delta_{max}$	$6\delta_{m}$

## Abbreviations

The following abbreviations are used in this manuscript:

PBV	Polyvinyl butyral
UD	Unidirectional (traction)
FPB	Four point bending
s.r.f.	Stress reduction factor
